# Identification of SARS-CoV-2 Variants of Concern Using Amplicon Next-Generation Sequencing

**DOI:** 10.1128/spectrum.00736-22

**Published:** 2022-06-27

**Authors:** Abdelmajeed Nasereddin, Hadar Golan Berman, Dana G. Wolf, Esther Oiknine-Djian, Sheera Adar

**Affiliations:** a Head of the Genomics Applications Laboratory, Core Research Facility, Faculty of Medicine, The Hebrew University of Jerusalem, Jerusalem, Israel; b Department of Microbiology and Molecular Genetics, Institute for Medical Research Israel Canada, Faculty of Medicine, Hebrew University of Jerusalem, Jerusalem, Israel; c Clinical Virology Unit, Hadassah Hebrew University Medical Center, Jerusalem, Israel; The Hebrew University—Hadassah School of Dental Medicine

**Keywords:** SARS-CoV-2, pandemic, PCR, mutations, WGS, NGS, variants of concern (VOC), variants of interest (VOI)

## Abstract

COVID-19 is caused by SARS-CoV-2, several virulent variants of which have emerged since 2019. More than 529 million people have been infected, and at least 6 million have died. Our aim was to develop a fast, accurate, low-cost method for detecting and identifying newly emerging variants of concern (VOCs) that could pose a global threat. The 341-bp DNA sequence of a specific region of the SARS-CoV-2’s spike protein was amplified by a one-step PCR on RNA samples from 46 patients. The product was sequenced using next-generation sequencing (NGS). DNA sequences from seven genomes, the original Wuhan isolate and six different representative variants obtained from the GISAID website, were used as references. Complete whole-genome sequences from local isolates were also obtained from the GISAID website, and their RNA was used for comparison. We used an amplicon-based NGS method (termed VOC-NGS) for genotyping and successfully identified all 46 samples. Fifteen (32.6%) were like the original isolate. Twenty-seven were VOCs: nine (19.5%) Alpha, eight (19%) Delta, six (14%) Beta, and four (8.7%) Omicron. Two were variants of interest (VOI): one (2%) Kappa and one (2%) Zeta. Two samples were mixtures of two variants, one of Alpha and Beta and one of Alpha and Delta. The Spearman correlation between whole-genome sequencing (WGS) and VOC-NGS was significant (*P* < 0.001) with perfect agreement (Kappa = 0.916) for 36/38 (94.7%) samples with VOC-NGS detecting all the known VOCs. Genotyping by VOC-NGS enables rapid screening of high-throughput clinical samples that includes the identification of VOCs and mixtures of variants, at lower cost than WGS.

**IMPORTANCE** The manuscript described SARS-Cov-2 genotyping by VOC-NGS, which presents an ideal balance of accuracy, rapidity, and cost for detecting and globally tracking VOCs and some VOI of SARS-CoV-2. A large number of clinical samples can be tested together. Rapid introduction of new mutations at a specific site of the spike protein necessitates efficient strain detection and identification to enable choice of treatment and the application of vaccination, as well as planning public health policy.

## INTRODUCTION

Viruses are relatively short sequences of nucleic acids that can mutate, giving rise to new genetic variants. This can occur either as single mutations or as combinations of mutations, and new variants can be distinguished from other existing viruses. Coronavirus disease (COVID-19) was originally detected in Chinese patients in late 2019 and was caused by what is now called severe acute respiratory syndrome coronavirus-2 (SARS-CoV-2). On 30 January 2020, the World Health Organization (WHO) declared the rapid spread of COVID-19 a pandemic and considered it a serious public health emergency of international concern (1, 2). Since then up to the time of writing of the manuscript (19 May 2022), the Worldometer website (3) has reported more than 525 million cases of covid-19 and more than 6 million deaths caused by the disease. While SARS-CoV-2 has a generally very low mutation rate as shown during the SARS-CoV-2 outbreak in the United States (4, 5), such high transmission rates have resulted in the emergence of many genetic variants across the world (6–9). SARS-CoV-2 is an RNA virus, believed to be evolved from Bat CoVs (10), belonging to the *Coronaviridae* family, *Coronavirinae* subfamily, *Betacoronaviruses* genus, and lineage 2B. The SARS-CoV-2 genome (~29 kb) consists of a positive-sense single-stranded RNA (+ssRNA), with a 5-cap and 3′ untranslated region (3′-UTR) poly(A) tail. The SARS-CoV-2 genome is stabilized by the nucleocapsid protein (N) and enveloped in a bilipid structure containing three main proteins: membrane protein (M), spike protein (S), and envelope protein (E). It has 14 open reading frames (ORFs) encoding different proteins, structural proteins (N, S, E, M), nonstructural proteins (nsps; ORF1 and ORF1ab), which are required for viral replication and assembly, and accessory proteins (ORF3, ORF4a, ORF4b, ORF5, ORF8). Whole-genome sequencing (WGS) studies showed several mutations, mainly single nucleotide polymorphisms (SNPs) and insertion/deletions (indels), that are mostly neutral or mildly deleterious (11).

The mutations giving rise to the variants are routinely monitored through sequence-based surveillance (9, 10). Of high priority are mutation and deletion within the spike (S) protein-coding sequence. The spike protein binds the ACE2 receptor (ACE2) of the host cell through its receptor-binding domain (RBD), which contains the receptor-binding motif (RBM) (12). This binding is the prerequisite for virus entry to the cells.

Mutations in the spike protein could affect both infectivity of the virus and the effectiveness of the current vaccines. For example, the D614G mutation that increased infectivity and stability of virions led to global transition from the original D614 genotype to the G614 (4). A hot spot region (HSR) has been defined for the RBD mutations of concern (i.e., L452R, E484K, N501Y) at the recruitment event (inferred by checking for the presence of RBD mutations shared within genomes closely related to the clade of interest) (11). It recognizes the human angiotensin-converting enzyme 2 (hACE2) with nanomolar affinity, triggering events that culminate with the fusion of the cellular and viral membranes (13). Mutations in this region participate in putative antibody escape variants. The RBD of SARS-CoV-2’s S1 domain strongly binds to both human and bat ACE2. SARS-CoV-2’s RBD is located between residues 331 to 524 or Thr333 to Gly526 (14) of the S1 domain, which enables it to bind to ACE2. It has been suggested that it binds ACE2 more strongly than SARS-CoV, which may be reflected in the higher infectiousness of SARS-CoV-2 compared to that of SARS-CoV (15). Increased infectivity of SARS-CoV-2 lineage B.1.1.7 is associated with the interaction force between the spike RBD Y501 mutant residue with the ACE2 receptor, which in this strain is increased (16). The most virulent mutations for immune escape are the ones within the RBD, e.g., K417N/T, N439K, L452R, Y453F, S477N, E484K, and N501Y (12). Furthermore, at least 10 mutations within this region enabled the Omicron variant to sweep the world with 3.8 million new cases reported in a single day (21 January 2022) (3).

SARS-CoV-2 variants have been classified by WHO into four categories (7, 8, 17): variant being monitored (VBM), variant of concern (VOC), variant of interest (VOI), and variant of high consequence (VOHC). Specific mutation markers can be used to identify variants: B.1.1.7 (Alpha, N501Y substitution), B.1.351 (Beta, combination of K417N, E484K, and N501Y substitution), P.1 (Gamma, combination of K417T, E484K, and N501Y substitutions), B.1.617.2 (Delta, combination of L452R and T478K substitution). More recently, the Omicron variant contains an extra 30 mutations in the spike region, including N440K, G446S, Q493R, G496S, Q498R, and Y505H. Specific mutations in VBMs and VOCs are primarily located in the distinct HSR within the spike. They have spread globally and were responsible for new surges of the pandemic (18, 19). VOIs are variants with specific genetic markers that are associated with a potential to influence the spread of SARS-CoV-2 by altering virus transmission, with antibody escape, and with affecting the efficacy of treatment (5, 8, 20–23). Two examples of mutations that have occurred in VOI are L452R, which is present in B.1.526.1, in Epsilon in both B.1.427 and B.1.429 lineages, and in B.1.617 (Kappa) lineages and sublineages, and E484K, seen in all B.1.525 (Eta) and P.2 (Zeta) variants but only in some strains of the B.1.526 (Iota) variant. Based on the CDC data available as of preparing the manuscript, there are no variants of SARS-CoV-2 that fall in the VOHC category (6, 17). In this study, VBMs and VOCs will be designated “VOC.”

A quick and accurate but low-cost surveillance system for detecting, identifying, tracking, and controlling the emergence of VOC variants is needed. Real-time identification of VOC and VOI will have a significant impact on the management of the current pandemic and on patient care (e.g., monoclonal antibody therapy) (24). Currently, applying real-time identification requires WGS capacity, which might not exist or might be restricted with regard to high-throughput capacity. WGS is also expensive and time-consuming. An accurate amplicon-specific target screening strategy would allow full screening for each positive sample. Several assays have been employed to detect only specific VOCs. For example, Aoki et al. (25) developed a genotyping platform for SARS-CoV-2 variants using high-resolution melting (HRM) analysis. Reverse transcription-quantitative PCR (RT-qPCR) assays, incorporating 14 primers and fluorescence-labeled probes, have also been developed for rapid detection of the SARS-CoV-2 mutations N501Y, 69 to 70del, K417N, and E484K, which occur in variants having a clinical impact (26). However, these technologies are specifically designed for previously identified variants. Here, we developed a quick, low-cost amplicon-based NGS method (termed VOC-NGS) that can maximize the testing of variants and broaden clinical laboratories’ ability to actively participate in identifying new VOCs.

## RESULTS

**FIG 1:**
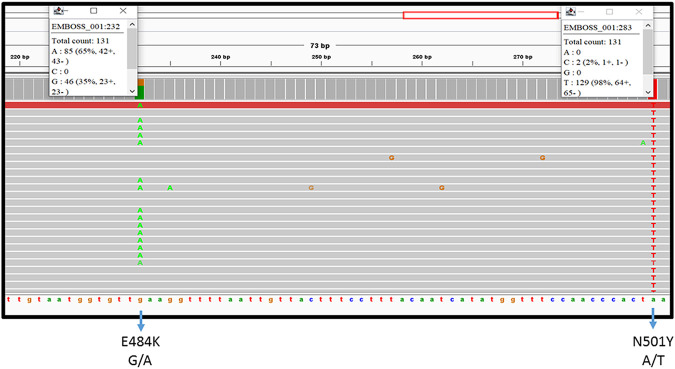
PCR amplification used in the study to obtain the two required fragments was done with a combination of three primers (MutF1, MutR1, and HS478F). 1: the optimized PCR using 0.5 μM of MutF1_MutR1 primers. In order to control the lower-molecular-weight product amplification, low concentration of 2nd forward primer HS478F (0.125 μM). 2: 0.5 μM used in all the primers sets.

The PCR amplification produced two fragments with the combination of three primers (Table 1), two forward and one reverse, and was selected to amplify the two nucleotide sequences seen as the bands 213 bp and 421 bp (Fig. 1). A lower concentration (0.125 μM compared to 0.5 μM) of the second forward primer, HS478F, was used to control excessive amplification of the lower-molecular-weight (213 bp) product, at the expense of the higher-molecular-weight product (421 bp).

**TABLE 1:** The primers used in the study and the final concentration of each one

Primer	Sequence 5′–3′	Final conc. μM	Product MW^*a*^ in bp
MutF1	TCGTCGGCAGCGTCAGATGTGTATAAGAGACAGGTCAGACAAATCGCTCCAGG	0.5	421
MutR1	GTCTCGTGGGCTCGGAGATGTGTATAAGAGACAGCTGGTGCATGTAGAAGTTCAAAAG	0.5	
HS478F	TCGTCGGCAGCGTCAGATGTGTATAAGAGACAGCTGAAATCTATCAGGCCGGTAGC	0.125	213

a MW, molecular weight.

**FIG 2:**
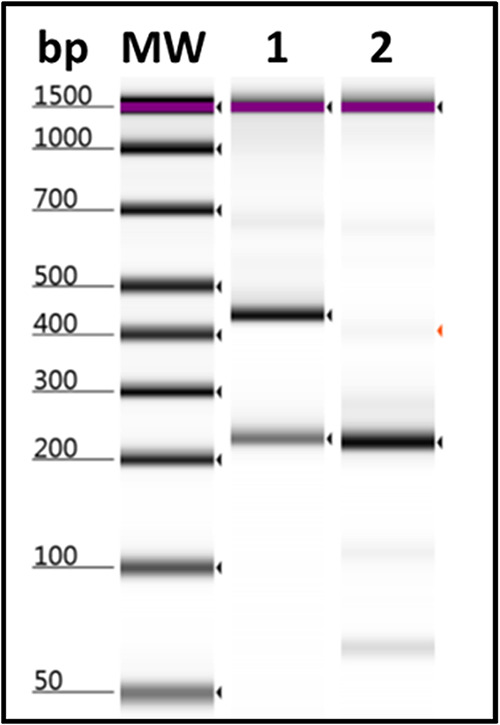
Multiple alignment of spike HSR DNA sequences from representative patients with COVID-19 and reference sequences for different VOCs from the GISAID website (Table 2). The 341-bp DNA sequence from the spike HSR region was aligned, using a free online multiple sequence alignment program (35). Representative clinical samples used in the figure are 848, 824, 829, 839, 835, and Sila. The red dots indicate those nucleotides identical to those of the DNA sequence of the Wuhan isolate. The blue and black letters present the changes that have occurred in the nucleotide sequence compared to that of the Wuhan isolate. The black/blue letters indicate the position of nucleotide substitution/deletion. The black N portions showed no sequenced region by the Nextseq machine since the sequence kit could not reach beyond 150 bp from one direction. Clearly, the VOC-NGS system was able to identify Omicron’s six unique mutations (N440K, G446S, Q493R, G496S, Q498R, and Y505H) in the target region. Ns: Any nucleotide.

VOC-NGS experiments were performed on RNA samples from 46 local patients, 10 of which were selected as they were known to contain the Alpha variant and the original strain. In parallel, and for comparison, WGS was performed. The WGSs of another 10 samples (Table 2) that were already in the GISAID website were used for comparison in assessing the efficacy of genotyping by VOC-NGS introduced here, which was able to identify all 46 (100%) samples studied. The number of variants found among the 46 samples studied were as follows: 15 (32.6%) were like the original isolate, 9 (19.5%) Alpha, 8 (19%) Delta, 6 (14%) Beta, and 4 (8.7%) Omicron. In addition, two VOIs were revealed, one (2.4%) Epsilon and one (2.4%) Zeta. Interestingly, two samples showed a mixture of different variants, one a mixture of Alpha and Beta variants (sample 824), the other a mixture of Alpha and Delta variants (sample 845). These mixtures were confirmed by WGS. The association of WGS and VOC-NGS was shown to be highly significant by Spearman correlation *P* < 0.001, which was applied to the 38 samples evaluated by both assays where 36 of 38 (94.7%) perfect agreements (Kappa = 0.916) occurred. There was 100% identity between the results of WGS and the genotyping by our VOC-NGS amplicon-based method. This method can detect any new mutation within this specific HSR. It can also reveal other VOIs such as Gamma (K417T, E484K, N501Y), Epsilon (L452R), and Zeta (E484K) (Fig. 2 and Table 2).

**TABLE 2:** The viral isolates used in this study, including the WGS mutations, lineages, and their mutations detected by VOC-NGS system

No. or GISAID	Sample barcode	*C_T_* ^*a*^	VOC-NGS	VOC-NGS mutations	WGS mutations	WSG linages	Collection date and location (mo.date.yr/city)
EPI_ISL_10550531	AbedN	NA	Omicron	N440K, G446S, Q493R, G496S, Q498R, Y505H	A67V, T95I, Q321E, G339D, R346K, S371L, S373P, S375F, K417N, N440K, G446S, S477N, T478K, E484A, Q493R, G496S, Q498R, N501Y, Y505H, T547K, D614G, H655Y, N679K, P681H, N764K, D796Y, N856K, Q954H, N969K, L981F	BA.1 21K	02.19.2022/Jerusalem
EPI_ISL_10550530	Sila	NA	Omicron	N440K, G446S, Q493R, G496S, Q498R, Y505H	A67V, T95I, G339D, R346K, S371L, S373P, S375F, K417N, N440K, G446S, S477N, T478K, E484A, Q493R, G496S, Q498R, N501Y, Y505H, T547K, D614G, H655Y, N679K, P681H, N764K, D796Y, N856K, Q954H, N969K, L981F	BA.1 21K	02.19.2022/Jerusalem
EPI_ISL_10550533	JER5	NA	Omicron	N440K, G446S, Q493R, G496S, Q498R, Y505H	A67V, T95I, G339D), S371L, S373P, S375F, K417N, N440K, G446S, S477N, T478K, E484A, Q493R, G496S, Q498R, N501Y, Y505H, T547K, D614G, H655Y, N679K, P681H, N764K, D796Y, N856K, Q954H, N969K, L981F	BA.1 21K	02.05.2022/Jericho
EPI_ISL_10550532	JER12	NA	Omicron	N440K, G446S, Q493R, G496S, Q498R, Y505H	A67V, V70I, T95I, Y145D, Q321Z, G339D, R346K, S371L, S373P, S375F, K417N, N440K, G446S, S477N, T478K, E484A, Q493R, G496S, Q498R, N501Y, Y505H, T547K, D614G, H655Y, N679K, P681H, N764K, D796Y, N856K, Q954H, N969K, L981F	BA.1 21K	02.05.2022/Jericho
EPI_ISL_2107518	AAS6	NA	Alpha	N501Y	A570D, D614G, D1118H, L5F, N501Y, P681H, S982A, T716I, V70I	B.1.1.7	10.24.2020/Jericho
EPI_ISL_1502573	AAS53	NA	Alpha	N501Y	A570D, D614G, D1118H, L5F, N501Y, P681H, S982A, T716I, V70I	B.1.1.7	05.02.2021/Salfit
EPI_ISL_2107510	AAS81	NA	Alpha	N501Y	A570D, D614G, D1118H, L5F, N501Y, N1098D, P681H, S982A, T716I, V70I	B.1.1.7	09.04.2021/Salfit
EPI_ISL_2107509	AAS80	NA	Alpha	N501Y	A570D, D614G, D1118H, F490S, L5F, N501Y, P681H, S982A, T716I, V70I	B.1.1.7	09.04.2021/Tubas
EPI_ISL_2107525	AAS77	NA	Alpha	N501Y	A570D, D614G, D1118H, L5F, N501Y, P681H, S982A, T716I, V70I	B.1.1.7	09.04.2021/Tubas
EPI_ISL_1273092	AAS29	NA	Original	Not detected	A520S, D614G	B.1.1.50	11.30.2020/Tubas
EPI_ISL_661272	WB-AQU-P3	NA	Original	Not detected	D614G	B	11.07.2020/Jericho
EPI_ISL_1273087	AAS20	NA	Original	Not detected	D614G	B.1.1.50	11.30.2020/Nablus
EPI_ISL_1502569	AAS48	NA	Original	Not detected	A570D, D614G, D1118H	NONE	02.05.2021/Nablus
EPI_ISL_1273095	AAS36	NA	Original	Not detected	D614G	B.1.1.50	11.30.2020/Jenin
96	2648372	NA	Epsilon	L452R	L18F, D80A, D215G, L242H, K417N, E484K, N501Y, D614G, A701V, A1020S, G1219C	B.1.351	02.09.2021/Jerusalem
249	2576218	NA	Beta	MIX E484K, N501Y	L452R, D614G, S929I, L1063F	B.1.362	01.25.2021/Jerusalem
416	2674042	NA	Zeta	E484K	L5F, P26S, T95I, D253G, E484K, D614G, A701V	B.1.526	04.25.2021/Jerusalem
610	225383	NA	Delta	L452R, T478K	T19R, T95I, E156G, L452R, T478K, D614G, P681R, D950N	B.1.617.2	05.? ?0.2021/Jerusalem
616	824	NA	Alpha_Beta	K417N, E484K, N501Y	H69S, E484K, N501Y, A570D, D614G, S640F, P681H, T716I, S982A, D1118H	B.1.1.7	05.12.2021/Jerusalem
641	2672172	NA	Delta	L452R, T478K	T19R, E156G, L452R, T478K, D614G, P681R, D950N	B.1.617.2	02.28.2021/Tel Aviv
721	S1	NA	Beta	K417N, E484K, N501Y	D80A, D215G, L242H, K417N, E484K, N501Y, D614G, A701V	B.1.351	10.03.2021/Jerusalem
722	S2	NA	Alpha	N501Y	N74K, N501Y, A570D, D614G, P681H, T716I, S982A, D1118H	B.1.1.7	10.? ?0.2021/Jerusalem
723	S3	NA	Original	Not detected	T95I, S477N, D614G, A831S	B.1.160	10.? ?0.2021/Jerusalem
724	S4	NA	Original	Not detected	S477N, D614G	B.1.160	10.03.2021/Jerusalem
725	S5	NA	Original	Not detected	S477N, D614G	B.1.160.16	10.? ?0.2021/Jerusalem
726	835	NA	Beta	K417N, E484K, N501Y	D614G	B.1	10.? ?0.2021/Jerusalem
727	839	NA	Original	Not detected	None	NA	10.? ?0.2021/Jerusalem
728	S8	NA	Beta	K417N, E484K, N501Y	V70F, D80A, D215G, L242H, K417N, E484K, N501Y, D614G, A701V	B.1.351	10.? ?0.2021/Jerusalem
729	S9	NA	Beta	K417N, E484K, N501Y		NA	10.03.2021/Jerusalem
730	829	NA	Alpha	N501Y	N501Y, A570D, D614G, P681H, T716I, S982A, D1118H	B.1.1.7	10.? ?0.2021/Jerusalem
731	S11	NA	Alpha	N501Y	N501Y, A570D, D614G, P681H, T716I, S982A, D1118H	B.1.1.7	10.? ?0.2021/Jerusalem
732	848	NA	Original	Not detected	S477N, D614G, A831S	B.1.160	10.? ?0.2021/Jerusalem
733	S13		Original	Not detected	S477N, D614G	B.1.160	10.03.2021/Jerusalem
734	S14		Original	Not detected	S477N, D614G, A831S	B.1.160	10.03.2021/Jerusalem
735	S15		Original	Not detected	N439K, D614G	B.1.258	10.03.2021/Jerusalem
736	S16		Original	Not detected	N74K, N501Y, A570D, D614G, P681H, T716I, S982A, D1118H	B.1.1.7	10.03.2021/Jerusalem
740	2667065	29	Delta	L452R, T478K	T19R, L452R, T478K, D614G, P681R, T791I, D950N	B.1.617.2	07.12.2021/Jerusalem
741	845	33	Alpha_Delta	L452R, T478K, N501Y	V3G, L5F, F140L, G142D, L242H, N501Y, A570D, D614G, P681H, T716I, S982A, D1118H	B.1.1.7	07.? ?0.2021/Jerusalem
742	2665422	15	Delta	L452R, T478K	T19R, E156G, L452R, T478K, D614G, P681R, D950N	B.1.617.2	07.13.2021/Jerusalem
743	2672449	15	Delta	L452R, T478K	L5F, T19R, E156G, L452R, T478K, D614G, P681R, D950N	B.1.617.2	07.? ?0.2021/Jerusalem
744	2665453	18	Delta	L452R, T478K	T19R, E156G, L452R, T478K, D614G, P681R, T791I, D950N	B.1.617.2	07.? ?0.2021/Jerusalem
745	2672564	22	Delta	L452R, T478K	T19R, E156G, L452R, T478K, D614G, P681R, D950N	B.1.617.2	07.16.2021/Jerusalem
746	2672568	20	Delta	L452R, T478K	T19R, E156G, L452R, T478K, D614G, P681R, D950N	B.1.617.2	07.16.2021/Jerusalem
747	2672569	35	Delta	L452R, T478K	Did not work due to high *C_T_*	NONE	07.16.2021/Jerusalem

a *C_T_*, threshold cycle; NA, not available.

**FIG 3:**
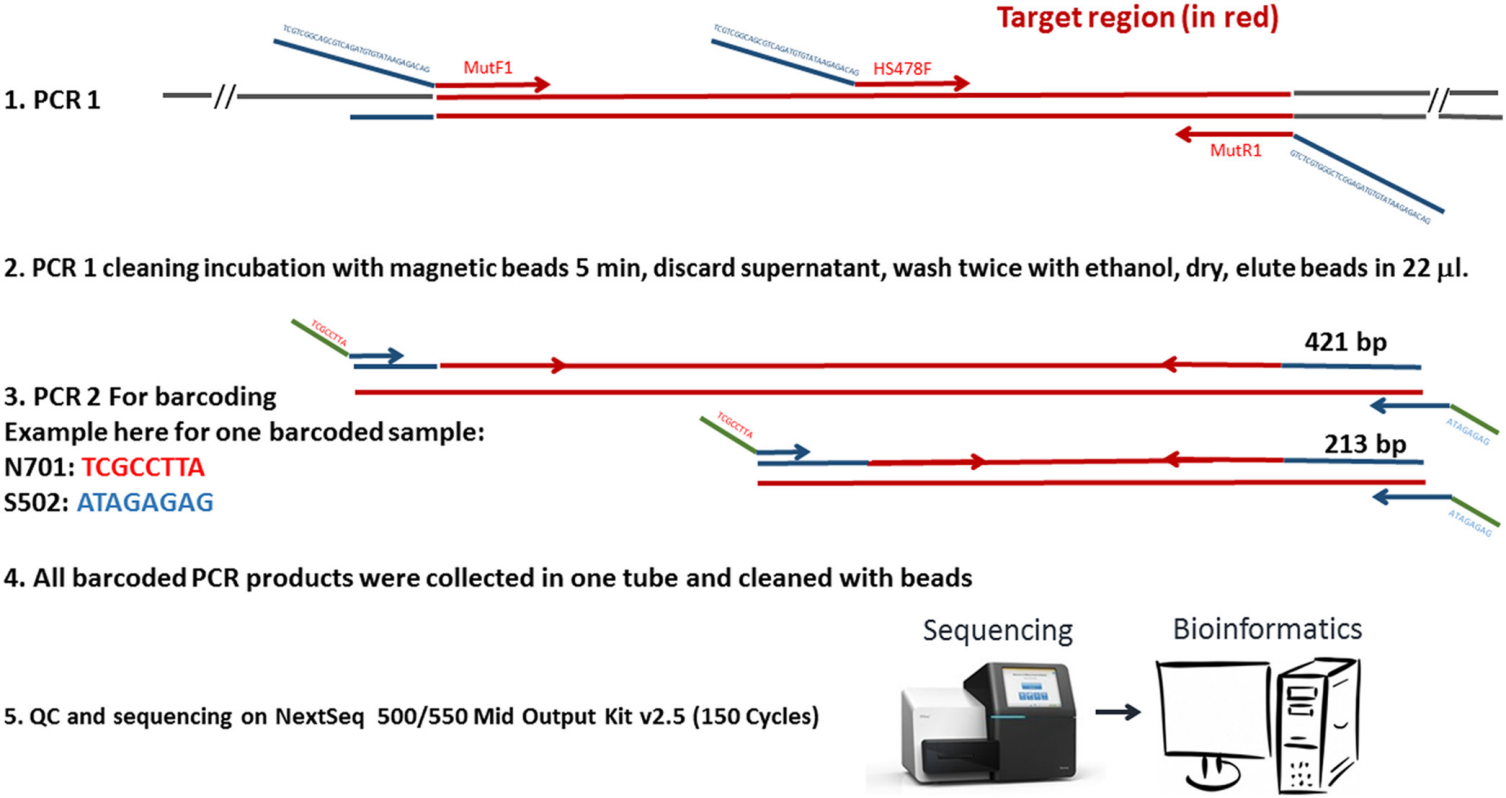
IGV results of galaxy mapping at positions 232 and 283, showing, respectively, the mutations E484K with the nucleotide G changed significantly to A at 65% coinfection-contamination and N501Y with the nucleotide A almost fully changed significantly to T at 98%.

Furthermore, genotyping by VOC-NGS was able to detect and quantify coinfection-contamination of different variants as shown by the clinical samples 824 (Alpha, 35%; Beta, 65%) and 845 (Alpha, 47%; Delta, 53%) in Fig. 3 where the percentages indicate the degree of coinfection-contamination. In Fig. 3, at position 283, sample 824 has the mutation N501Y with the nucleotide A changes to T at 98% coinfection contamination, and at position 232, sample 845 has the mutation E484K with the nucleotide G changed to A at 65% coinfection contamination (see the arrows below the chart and the box for quantification of the conversion at the top of the chart). The chart was displayed with local integrative genomics viewer (IGV).

## DISCUSSION

NGS was used in developing a new assay, VOC-NGS genotyping, for use in standard clinical laboratories to rapidly, reliably, and inexpensively screen for VOCs of SARS-CoV-2. This method is able to detect and identify the different spike protein mutations that have occurred in VOCs with a 94.1% agreement with identifications of local variants done by WGS. With a kappa value of 0.916, this is considered to be almost perfect agreement since a kappa value of between 0.81 and 1.00 is accepted as almost perfect agreement (27). VOC-NGS genotyping successfully identified the original isolates and the Alpha, Beta, Delta, and Omicron variants from the four consecutive surges of the COVID-19 pandemic in the region.

Several assays have been employed to detect and identify only VOCs, e.g., high-resolution melting (HRM) analysis to detect and identify variants of SARS-CoV-2 (25) and RT-qPCR assays to detect the mutations N501Y, 69 to 70del, K417N, and E484K occurring in variants of SARS-CoV-2 that have a clinical impact, using 14 sets of primers and fluorescence-labeled probes (26). Differences in annealing temperature can affect both assays. Both assays require a positive control for detecting the presence of each mutation and for building different HRM graphs. Also, both assays provide only limited information with regard to specific probes, e.g., the targeting of the N501Y mutation by its specific probes. Changes close to this mutation, a region known to be polymorphic, could prevent the probe’s annealing to the target site and consequently miss detection of this variant. The same goes for the HRM assay, where any change in the annealing temperature of the product component would cause it to change its melt curve and produce misleading peaks. Furthermore, appropriate positive controls will be needed for each SNP in each run. In contrast, genotyping by VOC-NGS is unbiased and provides entire sequences despite the occurrence of mutations within the product. If a mutation occurs at the target site where the primers anneal, a product will not be produced. Such events could be subject to follow-up investigation by longer PCR and Sanger sequencing. As genotyping by VOC-NGS is not based on specific probes and the need of doing WGS, it should enable early global tracking and monitoring of the spread of the known and new introduced coronavirus variants. Owing to its rapidity, accuracy, and high throughput, its wide employment would alert people in authority, policy makers and public health providers, to the emergence, spread, and reemergence of the virus and its variants, enabling them to try to prevent their spread.

There are advantages to using VOC-NGS genotyping, an amplicon-based NGS method, over WGS using the ARTIC v3 system (28). There is no need for amplification of the whole genome using two PCR pools, followed by Nextera DNA Flex preparation, which increases costs (ARTIC v3 sequencing costs £975 per 100 samples, using CoronaHiT-Illumina sequencing of the about 30,000 bp of the SARS-CoV-2 genome) (29). The total cost for genotyping by VOC-NGS is only £180 for 100 samples, which includes sequencing at a depth of 400× with a 150 MID kit costing £30, Luna one-step ready-mix costing £130, and Agencourt AMPure XP and Nextera barcodes costing £20. In total, this amounts to almost a fifth (18%) of the cost of Corona WGS (£180 versus £975).

While WGS provides more information on the virus and its variants, these fiscal calculations clearly show that detection and identification of variants of COVID-19 using genotyping by VOC-NGS costs less than that by WGS. Also, genotyping by VOC-NGS, which can be operated in different setups, is fast, as it produces cDNA and carries out specific HSR amplification with the specific primers in a single reaction. Moreover, free online analysis tools such as Galaxy, Genome Detective, and IGV produce accurate consensus sequences of the 341-bp region and can be employed by any standard laboratory performing data analysis, thus avoiding bioinformatic analysis costs. The products prepared from the sample for genotyping by VOC-NGS are suitable for use in all Illumina platform machines (MiniSeq, MiSeq, Nextseq, Iseq100i, and Novaseq machines) for 150-bp single and paired-end reads. For high-throughput screening, 384 samples can be loaded and run together owing to the barcode combinations of indices that can be multiplexed together, and the protocol is compatible with a robotic system.

Genotyping by VOC-NGS was able to identify all 341 bp of the spike HSR. This is where all the mutations giving rise to the known VOCs occur and encompasses the variants VOC Alpha 202012/01 GRY (B.1.1.7), first detected in the United Kingdom, VOC Beta GH/501Y.V2 (B.1.351), first detected in South Africa, VOC Gamma GR/501Y.V3 (P.1), first detected in Brazil/Japan, VOC Delta G/478K.V1 (B.1.617.2 plus AY.1 plus AY.2), first detected in India, and Omicron (B.1.1.529), first reported on 24 November 2021 in South Africa. In addition, the system was able to identify VOI Zeta GR/484K.V2 (P.2), first detected in Brazil, and VOI Epsilon G/452R.V3 (B.1.617.1), first detected in India.

However, the variants VOI Theta GR/1092K.V1 (P.3), first detected in the Philippines, and VOI Iota GH/253G.V1 (B.1.526), first detected in New York, USA, cannot be detected with genotyping by VOC-NGS, as their underlying mutations fall outside the HSR target zone. In this case, new PCR primers can be developed to detect and identify the mutations of new VOIs situated outside the HSR that can be included in the same PCR. Thereafter, the genotyping procedure would remain as described for VOCs. The cost of preparing these additional primers would be very low. The combination of primers used in the method described in this article is more straightforward in detecting and identifying VOCs and most VOIs and, therefore, has greater diagnostic utility than single-mutation-specific PCR approaches like the RT-qPCR and HRM assays.

The VOCs affect the distribution, expansion, and reemergence of the COVID-19 pandemic during its different surges and its disease severity and vaccine efficacy (12, 20, 21, 30). Genotyping by VOC-NGS is also able to detect and identify the Delta plus variant of SARS-CoV-2 recently discovered in Israel since the mutations K417T, E484K, and L452R are in the target region of the variant’s spike protein. VOC-NGS genotyping was able to reveal and quantify a mixture of two-variant infection as shown in two samples (824 and 845) in this study. This was previously reported in a rare case of coinfection of an unvaccinated elderly woman who was shown to be infected at the same time with the Alpha and Beta variants of SARS-CoV-2 (31). Furthermore, recent study on SARS-CoV-2 strains reveals potential contribution of coinfection with and recombination between different strains to the emergence of new strains (32).

To check for new mutations, the sampling of screen-negative specimens for WGS will be an important component of a full surveillance strategy, as the approach described here cannot predict the existence of new variants that carry new mutations. Addition of more amplicons by targeting new regions of interest for use in genotyping by VOC-NGS is a good option with no significant price difference.

This method could play a role in choosing therapy as an alternative to administering bamlanivimab and etesevimab, which are generally used to treat cases of COVID-19 but have shown reduced efficacy against the Beta variant B.1.351 that contains the mutations K417N plus E484K plus N501Y (23). Identifying these mutations will indicate the need for alternative therapy for patients infected with this variant (23, 30).

In cases of low RNA abundance (high RT-PCR cycles), the sensitivity of VOC-NGS is higher than that of NGS. This was apparent in clinical sample S747. This sample had threshold cycle levels (32), and failed in WGS, but was clearly identified by VOC-NGS genotyping. This is due to the targeting specific amplicons on the genome.

The technique’s main limitation remains the need of an NGS facility. However, this can be solved by shipping DNA libraries to either local or international companies, such as Macrogen, https://www.macrogen-europe.com/, and Novogene, https://en.novogene.com/, or laboratories with NGS capabilities.

In summary, genotyping by VOC-NGS presents an ideal balance of accuracy, rapidity, and cost for detecting and globally tracking VOCs and some VOIs of SARS-CoV-2. A large number of clinical samples can be tested together. Rapid introduction of new mutations at a specific site of the spike protein necessitates efficient strain detection and identification to enable choice of treatment and the application of vaccination, as well as planning public health policy.

## MATERIALS AND METHODS

### RNA samples.

**FIG 4:**
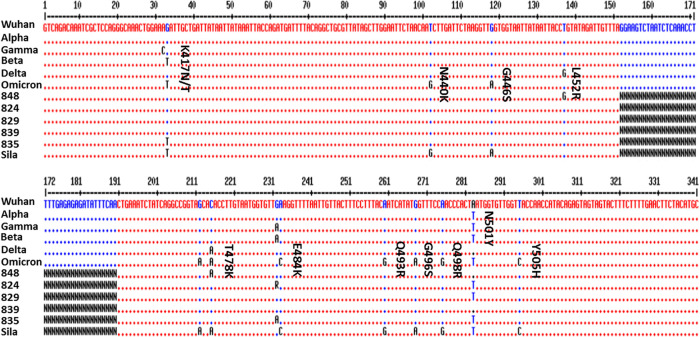
The target region for amplicon sequencing (341 bp) from the Wuhan isolate. Bold black arrows indicate the positions of the primers. Highlighted: sequences used for primers in this study. Bold red: the nucleotide conversions. Bold black font: the corresponding amino acid conversions. * indicates mutations found in the recent Omicron variant.

**FIG 5:**
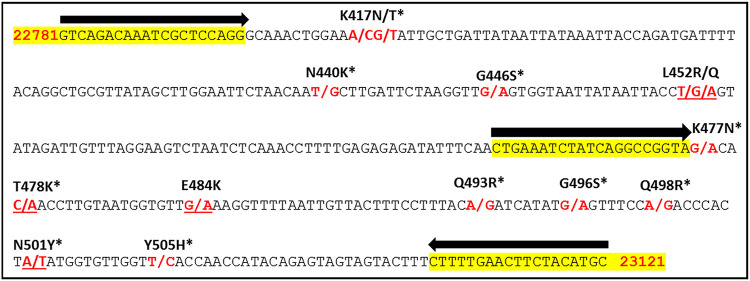
Schematic overview of the study workflow.

This study was part of the officially permitted diagnostic procedure of The Hebrew University Hadassah Medical Center; therefore, no additional approval from the Institutional Review Board was required. Samples were collected in Jerusalem between February and July 2021. Table 2 describes the clinical RNA samples and viral variants used in this study. The genetic sequence of the spike protein of SARS-CoV-2 was that of the Wuhan-Hu-1 isolate (GenBank accession number: NC_045512.2, 21563 to 25384). The nucleic acid sequence between the mutations K417N and N501Y was targeted as it encompasses all the mutations that have produced VOCs (Table 2 and Fig. 4). The schematic overview of workflow is shown in Fig. 5.

### Whole-genome sequencing of viruses from clinical samples.

RNA was extracted using the MagDEA Dx SV kit on the MagLEAD platform (Precision System Science Co., Ltd.) according to the manufacturer’s instructions. A volume of 280 μL lysis buffer was added to a 220-μL sample and eluted in 50 μL. The RNA concentration/quality was determined using a NanoDrop 1000 spectrophotometer (Thermo Fisher), and extracted RNA was kept at −70°C for further analysis.

Sequencing libraries were prepared using the Illumina COVIDSeq test and sequenced on Novaseq 6000 producing at least 3.3 million paired-end reads (150 nucleotides [nt]) per library. Library quality was analyzed using FastQC (version 0.11.8, Babraham Bioinformatics). Reads were aligned to the genome using Bowtie2 (version 2.3.4.3) with the command options -k 4 –no-discordant. Reads with more than 6 variants in 100 bases were discarded (single nucleotide variants [SNV], deletion or insertion each count as one variant). Variants were called using ivar variants (version 1.3.1). Consensus sequence was built based on the ivar variants table using the R Biostrings package according to the following rules. Positions with fewer than 10 reads were called N. Variants with frequency higher than 0.7 were included in the consensus sequence. Variants with frequency between 0.3 and 0.7 and at least 50 reads were considered “wobbles” using the IUPAC letters. Consensus sequences were submitted to Pangolin command-line tool (pangolin version 2.2.2 and pangoLEARN version 2021-02-12) and Nextclade (version 0.12.0) to determine the PANGO lineage and clade.

### PCR amplification of the HSR region.

Two sets of primers were designed, using the primer 3 online program, and synthesized. In accordance with the Nextera Illumina DNA library preparation kit (Illumina, San Diego, CA, USA), they included specific Illumina adaptor overhang sequences added at the 5′ end: TCGTCGGCAGCGTCAGATGTGTATAAGAGACAG for the forward primer and GTCTCGTGGGCTCGGAGATGTGTATAAGAGACAG for the reverse primer (Fig. 5).

PCR amplification to obtain the two required fragments was done with a combination of three primers (Table 1), two forward primers, MutF1 and HSF487, and one reverse primer, MutR1. This resulted in amplification of two nucleotide sequences (Fig. 1) detected as 213 bp (a 146-bp sequence flanked by 67 bp of primers HSF487 and MutR1) and 421 bp (a 354-bp sequence flanked by 67 bp of primers MutF1 and MutR1). To avoid an excess of the lower-molecular-weight product, a lower concentration of the second forward primer was used (0.125 μM HSF487 as opposed to 0.5 μM MutF1 and MutR1).

We conducted reverse transcription and PCR amplification as a one-step reaction that comprised cDNA preparation and PCR amplification, using the Luna Universal Probe one-step RT-qPCR kit (E3006, NEB) according to the manufacturer’s protocol. A total of 2.5 μL extracted RNA in a reaction volume of 20 μL was used. The PCR conditions were a reverse transcription cycle of 10 min at 55°C, then 1 min at 95°C, and then 40 cycles of 10 s at 95°C, together with annealing and extension in one step of 30 s at 60°C. PCR products were purified, using AMPureXP beads (Beckman Coulter, Brea, CA, USA). Twenty-five microliters of beads was added to the PCR product, incubated for 5 min at room temperature, and then put on a magnetic plate for 2 min, followed by two washes with freshly made 80% ethanol and then drying for 2 min on the plate. Finally, beads were eluted with 22 μL of elution buffer. The purified amplicons in a volume of 10 μL underwent eight cycles of PCRs to anneal dual-indexed barcodes with unique sequences: index 1 read 5′-CAAGCAGAAGACGGCATACGAGAT[i7]GTCTCGTGGGCTCGG, index 2 read 5′-AATGATACGGCGACCACCGAGATCTACAC[i5]TCGTCGGCAGCGTC. i7 and i5 are unique 8-bp sequences for identifying amplicons derived from each sample (Illumina, 2013). Then, 10 μL of each sample was pooled and purified again with AMPureXP beads. The purified pool product was adjusted to 4 nM and sequenced with a NextSeq 500/550 mid output kit v2.5 (150 cycles), using a NextSeq 500 machine (Illumina, San Diego, CA, USA), aiming at ~1,000 reads giving a depth of 400× per sample. Binary base call (BCL) output files from a Nextseq 500 machine were converted to FASTQ format, using BCL to FASTQ (bcl2fastq v2.20.0.422, Illumina, Inc.). FASTQ format files were analyzed using the Galaxy program (Galaxy version 0.7.17.1) (https://usegalaxy.eu/) (33). The 341-bp DNA sequence was obtained from the sequencing of two PCR products. The first 150-bp DNA sequence was from read 1 (forward primer 1) from PCR1, followed by a gap of 40 Ns, and then the second 150-bp sequence started from forward primer 2 (read 1) from PCR2 (see Fig. 2). The 341-bp consensus sequences were obtained by mapping reads with BWA-MEM map medium and long reads (>100 bp) against the Wuhan-Hu-1 SARS-CoV-2 reference isolate (selected 341 bp) fragment from isolate hCoV-19/Wuhan/Hu-1/2019, GenBank accession number NC_045512, GISAID accession ID EPI_ISL_402125. The mapping was displayed with a local integrative genomics viewer (IGV), and the consensus sequence from the IGV was copied and accepted as the sequence of the sample’s variant. The genome detective virus tool version 1.133 (https://www.genomedetective.com/app/typingtool/virus/) was used to directly reveal the spike mutation at the DNA and amino acid levels (34). A free online multiple sequence alignment program (35) was used to align these HSR DNA sequences of the spike protein from patients with COVID-19 and different VOCs from the GISAID website (Fig. 4 and Table 2).

### Statistical analysis.

The Statistical Package for the Social Sciences (SPSS) computer software program version 15.0 (SPSS Inc.) was used to test the Spearman correlation as a nonparametric test used to measure the degree of association between VOC-NGS assay and the WGS assay results. The free online GraphPad (https://www.graphpad.com/quickcalcs/kappa1/?K=5) program was used for kappa agreement tests (27). Cohen’s kappa coefficient (κ) is a measure of the agreement between two tests beyond that expected by chance, where 0 is chance agreement and 1 is perfect agreement for the VOC-NGS assay performance against the WGS method.

### Data availability.

The original data using a next-generation sequencing data set from the GISAID website used in the study are found in Table 2. The data sets used and/or analyzed during the present study are available from the corresponding author on reasonable request.
